# Patient Experience of Primary Care in One Small Hospital and One Non‐Bedded Clinic in a Mountainous Rural Area of Japan

**DOI:** 10.1002/jgf2.70156

**Published:** 2026-08-06

**Authors:** Nobuaki Ohara, Masahiko Koda, Mikio Takechi, Kazuhiro Takemoto, Mikako Hirai, Hiroyuki Kato, Yuma Otsuka, Shin‐ichi Taniguchi, Daisuke Son

**Affiliations:** ^1^ Department of Internal Medicine National Health Insurance Yodakubo Hospital Nagano Japan; ^2^ Department of Internal Medicine Hino Hospital Tottori Japan; ^3^ National Health Insurance Ebi Clinic Tottori Japan; ^4^ Department of Second Internal Medicine, Faculty of Medicine Tottori University Yonago Japan; ^5^ Department of Gastroenterology National Hospital Organization Yonago Medical Center Yonago Japan; ^6^ Department of Internal Medicine National Health Insurance Chizu Hospital Tottori Japan; ^7^ Department of Community‐Based Family Medicine, Faculty of Medicine Tottori University Yonago Japan

**Keywords:** Japan, patient experience, primary care, primary care assessment, rural healthcare

## Abstract

**Background:**

In Japan, both hospitals and outpatient clinics serve as primary care providers. However, the characteristics of primary care practices in hospitals and clinics, as well as the role and functions of primary care in mountainous and rural areas, are poorly understood. The purpose of this study was to identify the role and functions of primary care in small hospitals and outpatient clinics in mountainous areas, as well as the characteristics of primary care practices specific to these regions.

**Methods:**

A descriptive study was conducted between January and March 2024 at two municipal facilities in western Tottori Prefecture: Hino Hospital and Ebi Clinic. Adult patients who had received care in general medicine or general practice for more than six months were invited to complete the Japanese version of the Primary Care Assessment Tool (JPCAT).

**Results:**

Valid responses were obtained from 254 patients at Hino Hospital and 27 at Ebi Clinic, with response rates of 90.6% and 100%, respectively. First Contact scores were higher at Hino Hospital, while Community Orientation scores were higher at Ebi Clinic. Other domain scores showed broadly similar ranges.

**Conclusions:**

Differences in patterns of First Contact and Community Orientation were observed across the two facilities. These findings describe how primary care is perceived within a shared rural context and should not be interpreted as causal or generalizable differences between facility types.

## 1 Introduction

In Japan, residents are encouraged to have a designated primary care physician to improve healthcare coordination and manage rising medical costs. However, the country operates a free‐access healthcare system, allowing individuals to visit any medical institution without a referral. To address this apparent contradiction, the Japan Medical Association broadly defines a “primary care physician” as any doctor who serves a gatekeeping function, regardless of the facility type [1].

This definition aligns with primary care models in Western countries, where primary care physicians work in outpatient clinics as gatekeepers to guide patients through the healthcare system [2]. A national survey reported that 73.1% of Japanese respondents considered themselves to have a “primary care physician”, with minimal differences between urban and rural areas. Among those physicians, 82.1% were affiliated with outpatient clinics, 15.9% with small‐ to medium‐sized hospitals (< 200 beds), and 7.7% with large hospitals (> 200 beds) [3].

In practice, however, many primary care physicians in Japan do not fully perform the gatekeeping function. Patients often bypass primary care and seek specialist services directly at large hospitals, which leads to overcrowded outpatient departments and inefficient care delivery [4, 5]. In response, there has been renewed attention to the importance of primary care physicians' gatekeeping roles.

To assess the quality and functions of primary care, the Primary Care Assessment Tool (PCAT) was developed in the United States. The Japanese version (JPCAT) was adapted to reflect the characteristics of Japan's healthcare system and is the first validated patient experience (PX) instrument for comprehensively evaluating primary care performance in Japan [6].

Previous studies comparing patient experiences between outpatient clinics and hospitals in Japanese urban areas have shown that outpatient clinics have higher patient satisfaction and that negative healthcare experiences among older adults are associated with social isolation [7, 8].

In rural areas, differences from urban areas include limited access to medical care and difficulties in seeing specialists [9]. The strong sense of community ties has also been noted as potentially enhancing trust in healthcare providers [10, 11]. Healthcare experiences in rural areas are shaped by unique social and geographic contexts, making quantitative evaluation difficult [12].

In Japan, few studies have quantitatively examined primary care physician functions in rural areas, particularly in small hospitals and outpatient clinics. Research focusing on mountainous regions and comparisons with urban areas remains limited. Therefore, we decided to conduct a descriptive survey focusing on the functions of walk‐in clinics and small hospitals in mountainous and rural areas.

The two study sites—Hino Hospital and Ebi Clinic—are located in mountainous areas of western Tottori Prefecture and share similar geographic, demographic, and social characteristics. While their organizational structures differ, both institutions serve aging rural populations within the same regional context.

By descriptively comparing patient experiences across these two facilities within a single mountainous area, this study seeks to clarify how healthcare is perceived and experienced in rural settings, rather than to frame institutional differences as competitive or oppositional. This study aims to describe the characteristics of two different primary care models in a single mountainous area, rather than comparing their superiority.

## 2 Methods

### 2.1 Participants and Time Period

This study was conducted at Hino Hospital and Ebi Clinic, two municipally operated healthcare facilities in western Hino County, Tottori Prefecture, Japan. Hino Hospital is located in Hino Town, while Ebi Clinic is in Kofu Town. As of May 1, 2023, Hino County had a population of 8920, with an aging rate of 52.3% (i.e., the proportion of residents aged 65 or older). Approximately 88% of the county is forested, indicating a predominantly mountainous landscape.

Hino Hospital is staffed by five full‐time internists, one general surgeon, one orthopedic surgeon, and one ophthalmologist. In addition, a primary care physician from the Department of Community Medicine at Tottori University is dispatched to provide daily inpatient and outpatient care. A specialist from the Department of Specialized Medicine at Tottori University also visits weekly to offer outpatient consultations.

Ebi Clinic is staffed by one full‐time internist and one primary care physician. Like Hino Hospital, it receives weekly outpatient support from three specialty departments at Tottori University.

The survey was conducted between January and March 2024. Questionnaires were distributed to patients who regularly visited either of the two facilities during this period.

Eligible participants were adults aged 18 years or older who had been receiving care in general medicine or general practice for more than six months at either facility. Patients were excluded if they were under 18 or had dementia that impaired their ability to understand the questionnaire. For patients with physical disabilities (e.g., limb loss) but preserved cognitive function, family members were permitted to assist with questionnaire completion.

### 2.2 Study Design

This was an observational cross‐sectional study using a self‐administered questionnaire. Healthcare staff distributed printed questionnaires to patients during their routine visits. Respondents completed the form while waiting for their consultation and submitted it anonymously into a sealed collection box, which ensured that staff could not view the responses.

### 2.3 Questionnaire Items

#### 2.3.1 Socio‐Demographic Characteristics

The questionnaire included items on gender, age, level of education, annual household income, history of visits to the medical institution, primary department visited, number of comorbidities, and distance from the respondent's residence to the facility. These items were developed with reference to previous studies.

#### 2.3.2 Japanese Version of the Primary Care Assessment Tool (JPCAT)

The primary outcome measure of this study was the total score from the Japanese version of the Primary Care Assessment Tool (JPCAT), developed by Aoki et al. [6] The JPCAT is the first validated instrument in Japan designed to assess the quality of primary care from the patient's perspective. It evaluates six core domains: first contact (accessibility), longitudinality (continuity), coordination, comprehensiveness (services available), comprehensiveness (services provided), and community orientation.

### 2.4 Statistical Analysis

Patient characteristics were analyzed based on responses collected through the JPCAT questionnaire. Each item in the JPCAT was rated on a five‐point Likert scale, with scores converted to a 0–4 scale. For each domain, the mean item score was multiplied by 25 to generate a score ranging from 0 to 100, where higher scores indicate better patient experiences. The average score for each domain was calculated separately for each facility. To examine variations in score distributions, we conducted a between‐group test as a supplementary analysis.

### 2.5 Ethical Considerations

The questionnaire was completed anonymously, and measures were taken to ensure that individual respondents could not be identified. Informed consent was obtained at the time of questionnaire distribution, with a clear explanation that participation was voluntary and that participants could withdraw at any time. The study protocol was approved by the Ethics Committee of Hino Hospital (Approval No. 2023‐3).

## 3 Results

A total of 254 responses were collected from Hino Hospital and 27 from Ebi Clinic. The valid response rates were 90.6% and 100%, respectively. In both groups, the male‐to‐female ratio was approximately 1:1.

The most common age group at Hino Hospital was 70–79 years, while at Ebi Clinic it was 80–89 years. In both settings, the majority of respondents reported an annual household income of less than 2.99 million yen. Additionally, most participants in both groups had been receiving care at the respective facility for over 10 years.

Regarding geographic access, 24.0% of respondents at Hino Hospital lived more than 10 km from the facility, whereas all respondents at Ebi Clinic lived within 10 km (Table 1).

**TABLE 1:** Socio‐demographic and clinical characteristics of patients by medical institution.

	Total (*N* = 281)	Hino Hospital (*N* = 254)	Ebi Clinic (*N* = 27)
*Sex, N (%)*			
Male	127 (45.2)	114 (44.9)	13 (46.4)
Female	146 (52.0)	132 (51.9)	14 (51.9)
Data missing	8 (2.8)	8 (3.2)	0 (0)
*Age, N (%)*			
> 59	24 (8.5)	23 (9.1)	1 (4.2)
60–69	55 (20.0)	51 (20.0)	4 (14.8)
70–79	110 (39.1)	102 (40.2)	8 (29.6)
80–89	71 (32.6)	59 (23.2)	12 (44.4)
< 90	10 (3.6)	8 (3.2)	2 (7.4)
Data missing	11 (3.9)	11 (4.3)	0 (0)
*Education, N (%)*			
> Junior high school	53 (18.9)	45 (17.7)	8 (29.6)
High school	169 (60.1)	152 (59.8)	17 (62.3)
Junior college	16 (5.7)	16 (6.3)	0 (0)
< College	30 (10.7)	28 (11.0)	2 (7.4)
Data missing	13 (4.6)	13 (5.1)	0 (0)
*Annual Household Income (million yen), N (%)*			
< 2.99	117 (41.6)	105 (41.3)	12 (44.4)
3.00–4.99	60 (21.4)	50 (19.7)	10 (37.0)
5.00–6.99	25 (8.9)	25 (9.8)	0 (0)
7.00–9.99	7 (2.5)	5 (2.0)	2 (7.4)
> 10	13 (4.6)	12 (4.7)	1 (4.2)
Data missing	58 (20.6)	56 (22.0)	2 (7.4)
*History of each medical institution visits (year), N (%)*			
< 1	8 (2.8)	8 (3.2)	0 (0)
1–5	65 (23.1)	63 (24.8)	2 (7.4)
6–10	41 (14.6)	41 (16.1)	0 (0)
> 10	146 (52.0)	123 (48.4)	23 (85.2)
Data missing	16 (5.7)	16 (6.3)	0 (0)
*Number of regularly consultation department, N (%)*			
1	120 (42.7)	106 (41.7)	14 (51.6)
2	76 (27.0)	71 (27.9)	5 (18.5)
> 3	71 (25.3)	65 (25.6)	6 (22.2)
Data missing	16 (5.7)	12 (4.7)	2 (7.4)
*Number of comorbidities, N (%)*			
1	108 (38.4)	98 (38.6)	10 (37.0)
2	78 (27.8)	71 (28.0)	7 (25.9)
> 3	57 (20.3)	49 (19.3)	8 (29.6)
Data missing	46 (16.4)	42 (16.5)	2 (7.4)
*Distance from each medical institutions (km), N (%)*			
> 1	41 (14.6)	35 (13.8)	6 (22.2)
1–5	57 (20.3)	45 (17.7)	12 (44.4)
5–10	88 (31.3)	82 (32.3)	6 (22.2)
> 10	61 (21.7)	61 (24.0)	0 (0)
Data missing	13 (4.6)	13 (5.1)	0 (0)

*Note:* Values are presented as number and percentage. The straight‐line distance from representative residential locations to each facility was calculated using Google Maps.

**FIGURE 1:**
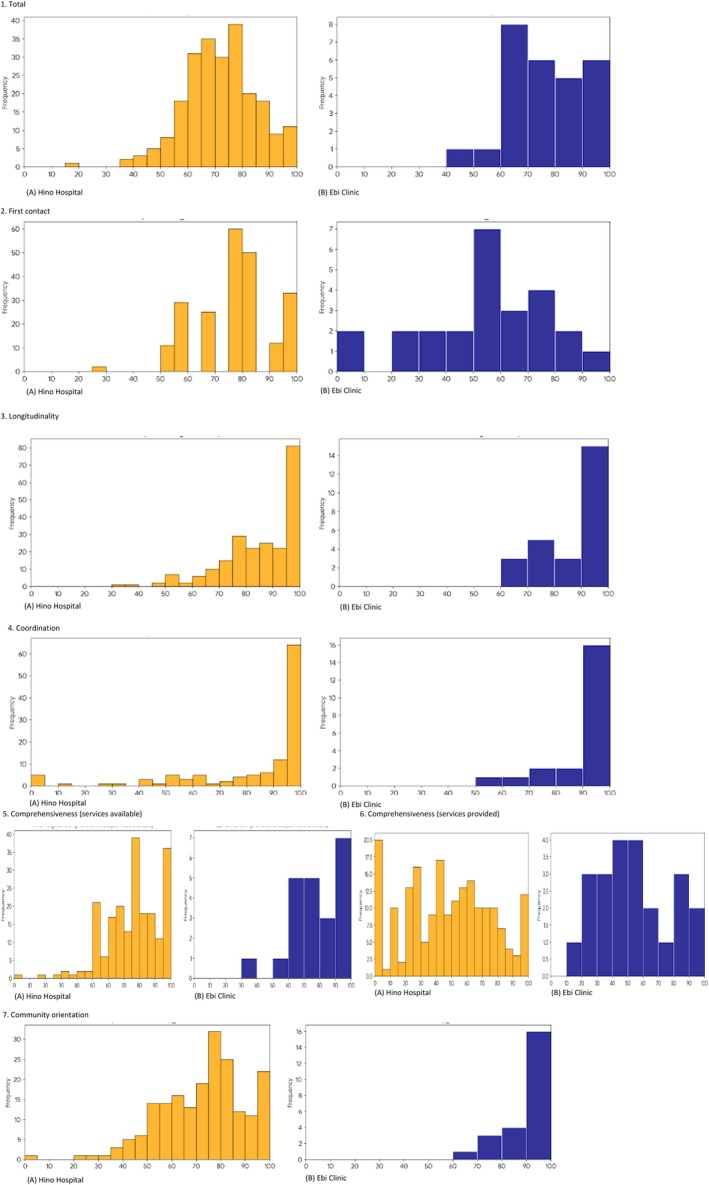
Distribution of JPCAT domain scores at Hino Hospital and Ebi Clinic. Each panel displays histograms of patient‐reported scores for each JPCAT domain, with Hino Hospital shown on the left and Ebi Clinic on the right. The horizontal axis (*x*‐axis) represents the JPCAT domain score on a fixed scale from 0 to 100, calculated using the standard JPCAT scoring method, and an identical bin width is applied to both facilities so that the distributions can be read on a common scale. The vertical axis represents the number of respondents. A bimodal distribution is evident in the Longitudinality domain at both facilities, indicating variation in perceived continuity of care. Considerable variance is also observed in the Comprehensiveness domains, particularly at Ebi Clinic. Because the number of respondents differs between the two facilities (Hino Hospital, *n* = 254; Ebi Clinic, *n* = 27), the vertical‐axis (frequency) scale differs between the Hino Hospital and Ebi Clinic histograms, whereas the horizontal‐axis (score) range and bin width are identical for both facilities.

The JPCAT domain scores are summarized in Table 2. The First Contact score was higher at Hino Hospital (76.7) than at Ebi Clinic (54.7). In contrast, Community Orientation was higher at Ebi Clinic (89.6) than at Hino Hospital (70.6). Other domain scores showed similar ranges across the two facilities. Histograms of each JPCAT domain are shown in Figure 1. At Hino Hospital, the distributions for First Contact, Comprehensiveness (Available Services), Comprehensiveness (Services Provided), and Community Orientation were generally normal. In contrast, the scores obtained in the Comprehensiveness domain at Ebi Clinic showed variation.

**TABLE 2:** JPCAT domain scores of medical institutions.

	Total (*N* = 257)	Hino Hospital (*N* = 230)	Ebi Clinic (*N* = 27)	*p*
Total	72.21 (13.30)	71.71 (13.21)	76.44 (13.54)	0.112
First contact	74.45 (17.27)	76.68 (14.81)	54.66 (24.19)	< 0.001
Longitudinally	84.08 (14.71)	83.82 (14.82)	86.35 (13.90)	0.439
Coordination	83.39 (24.78)	82.04 (26.12)	90.68 (14.00)	0.150
Comprehensiveness (services available)	74.04 (18.07)	73.65 (18.08)	77.73 (18.04)	0.352
Comprehensiveness (services provided)	46.27 (27.72)	45.53 (28.11)	52.61 (23.74)	0.276
Community orientation	72.68 (18.17)	70.61 (17.79)	89.58 (11.22)	< 0.001

*Note:* Scores are presented as mean (standard deviation). The Mann–Whitney *U* test was used to compare scores between groups.

At both Hino Hospital and Ebi Clinic, a bimodal distribution was observed in the Continuity domain, indicating the presence of two distinct patient groups. And, in the Coordination domain, while most patients scored highly, a small number of low scores were also observed.

## 4 Discussion

This study yielded several key findings: The overall JPCAT score across the two facilities was 72.2. The highest‐rated domains were Longitudinality (84.1) and Coordination (83.4). The First Contact score was higher at Hino Hospital (76.7) than at Ebi Clinic (54.7). In contrast, Community Orientation was higher at Ebi Clinic (89.6) than at Hino Hospital (70.6). Comprehensiveness was low at both facilities (Hino Hospital: 45.5, Ebi Clinic: 52.6).

We selected the study by Aoki et al. [13] as representative of urban data. Although their study included both urban and rural data, the majority of participating facilities—both small‐to‐medium‐sized hospitals and non‐bed clinics—were located in urban areas. Therefore, we determined that the urban data were predominantly represented in their findings.

### 4.1 Overall Score and Comparison to Urban Data

The overall JPCAT score in this study (72.2) was comparable to scores reported in urban primary care settings by Aoki et al. [13] This shows that, despite geographical and demographic differences, the perceived quality of primary care in mountainous areas can be maintained at levels similar to those in urban regions.

### 4.2 First Contact (Proximity)

The First Contact score in this study (74.5) was higher than that reported by Aoki et al. [13] (50.6). Previous surveys of older adults in rural Japan have identified “psychological connection,” “a sense of being cared for,” and “security” as core components of access, which may have contributed to the higher proximity score observed [14]. As an unmeasured factor, mountainous residents in this study may have perceived stronger psychological proximity and accessibility to their local facilities.

### 4.3 Longitudinality and Coordination

Scores for Longitudinality (84.1) and Coordination (83.4) were higher than those in Aoki's study (Longitudinality: 79.8; Coordination: 70.2) [13]. These results are notable given that rural areas often face structural limitations such as reduced access to specialists and limited after‐hours care.

In this study, rural areas scored highly on the coordination score. This reflects a characteristic of Hino Hospital, where many patients visit multiple departments (two or more departments: 53.5%).

As unmeasured factors, the high Longitudinality score shows that patients in mountainous regions experience greater continuity in their relationships with primary care providers. Two contextual factors may explain this. First, rural facilities often serve as long‐term care providers, resulting in more stable doctor–patient relationships [15, 16]. Second, patients in these settings place greater value on familiarity and trust, consistent with previous research indicating that continuity is especially valued in geographically isolated environments [17].

### 4.4 Community Orientation

The Community Orientation score in this study (72.7) showed a lower tendency than that in Aoki et al.'s findings (74.0) [13]. This may reflect a characteristic of Hino Hospital, where many patients travel long distances (10 km or more: 24.0%) to receive outpatient care in mountainous and rural areas. Furthermore, at both Hino Hospital and Ebi Clinic, the histogram for Community Orientation exhibited a bimodal distribution. This suggests that there are patients who are satisfied with the healthcare facilities' community activities as well as those who are dissatisfied.

### 4.5 Comprehensiveness

The comprehensiveness score of the services provided (46.3) tended to be lower than those reported in previous studies [13]. This suggests ongoing limitations in the perceived scope of care, even when Comprehensiveness (Services Available) was high (74.0). The gap between perceived availability and actual service provision may reflect issues such as communication breakdowns, inconsistent delivery, or varying patient expectations in rural communities. The results of this study did not reveal the reasons for the decline in comprehensiveness (services provided). One hypothesis is that, due to a shortage of human resources at medical institutions in mountainous and rural areas, staff at both facilities were forced to focus on overt medical problems. Further investigation is needed on this point.

### 4.6 Characteristics of Hino Hospital and Ebi Clinic

This study identified the distinctive characteristics of Hino Hospital and Ebi Clinic, suggesting that while general primary care quality may be comparable, the specific roles of primary care physicians differ by facility type. These findings suggest that the relationship between Hino Hospital and Ebi Clinic is not adversarial but complementary.

In this study, First Contact (Proximity) scores were higher at Hino Hospital. One possible explanation is that patients may associate small hospitals with a wider range of medical services, including 24‐h emergency care, which may enhance their perceived accessibility. Previous research has indicated that proximity is influenced by institutional factors such as service range, facility reputation, and the perceived expertise and qualifications of physicians [18].

Histogram analysis (Figure 1) revealed a bimodal distribution in the Longitudinality domain at both facilities, suggesting two distinct subgroups: patients who perceived a strong sense of continuity and those who did not. This variability may be related to differences in frequency of visits, consistency of providers, or the nature of the physician–patient relationship.

In contrast, the higher Community Orientation score at the non‐bedded clinic may reflect its deeply integrated role within the local community. A prior report suggests that strong community orientation in rural clinics is supported by factors such as continuity of care by a single physician, service delivery tailored to local needs, effective coordination of healthcare resources, and timely referral to specialists [19]. Although this factor was not investigated in the present study, the higher community orientation score may have been influenced by the individual director who has worked for many years at the non‐bedded clinic.

Several prior studies have demonstrated that the care environment has a significant impact on patient experience in primary care [20, 21, 22]. For example, a cross‐sectional study comparing 13 hospitals and non‐bedded clinics in Japan found that First Contact (Proximity) scores were higher in the hospital group, and that Community Orientation was also higher in hospitals than in clinics [13].

At the same time, the particularly high Community Orientation score observed at Ebi Clinic may be influenced by its unique patient experience. To better understand the factors contributing to this result, future research could incorporate qualitative methods to explore patients' perspectives at Ebi Clinic in greater depth.

Hino Hospital and Ebi Clinic exist within the same region, but it was shown that the roles each facility plays differ. Hino Hospital scored high in First Contact, and many of its patients traveled from distant locations or visited two or more departments. In contrast, Ebi Clinic scored high in Community Orientation, with many patients having a history of visits spanning over 10 years and coming from nearby areas. In other words, Hino Hospital appeared to provide greater accessibility through its numerous departments, while Ebi Clinic maintains continuity and a community‐oriented focus.

Importantly, these findings indicate that primary care in rural settings should be understood as a system composed of interdependent components rather than isolated facilities. The relationship between Hino Hospital and Ebi Clinic appears to be complementary rather than competitive, forming an integrated care system adapted to local conditions. This perspective aligns with the concept of context‐sensitive primary care, which emphasizes the importance of tailoring healthcare delivery to local needs and resources.

From a policy perspective, these results suggest that improving primary care quality in rural areas requires strengthening the coordination between hospitals and clinics rather than applying uniform standards derived from urban models. Enhancing community orientation in hospitals and improving accessibility in clinics may contribute to optimizing the overall system.

To our knowledge, this is the first study to evaluate the functions of primary care physicians in both small hospitals and non‐bedded clinics located in mountainous regions, using a validated patient experience–based instrument. The high response rates at Hino Hospital (90.6%) and Ebi Clinic (100%) reduce the risk of selection bias. Moreover, the use of the JPCAT—a psychometrically validated adaptation of the internationally recognized Primary Care Assessment Tool (PCAT)—enhances the methodological rigor of the study.

However, this study has several limitations that warrant consideration.

First, the small sample size at Ebi Clinic (*n* = 27) may have introduced imbalances or latent heterogeneity. Second, as the study was conducted at only two facilities within a single region, the generalizability of our findings is limited. Third, since we did not adjust for potential confounders and relied solely on univariate analyses, the results may have been influenced by confounding factors. Finally, statistical analyses were performed for descriptive purposes only; therefore, these results should be interpreted with caution and not as formal inferential comparisons.

## 5 Conclusion

Overall, both the small hospital and the non‐bedded clinic in this mountainous region showed high JPCAT scores, particularly in the domains of Longitudinality and Coordination. Differences in patterns of First Contact and Community Orientation were observed across the two facilities.

These findings describe how primary care is perceived within a shared rural context and should not be interpreted as causal or generalizable differences between facility types. The observed patterns may reflect contextual and unmeasured factors specific to each facility.

As this study was conducted in only two facilities within a single region, the findings are not generalizable. Further descriptive studies involving multiple institutions in diverse mountainous areas are needed to better understand the characteristics of primary care in rural Japan.

## Author Contributions

**Mikio Takechi:** investigation. **Masahiko Koda:** supervision, conceptualization, methodology, writing – review and editing. **Nobuaki Ohara:** conceptualization, investigation, writing – original draft, project administration, writing – review and editing, methodology. **Mikako Hirai:** investigation. **Kazuhiro Takemoto:** investigation. **Hiroyuki Kato:** investigation. **Yuma Otsuka:** investigation. **Shin‐ichi Taniguchi:** writing – review and editing, methodology, supervision. **Daisuke Son:** software, formal analysis, data curation, supervision, writing – review and editing, methodology, conceptualization.

## Funding

The authors have nothing to report.

## Conflicts of Interest

The authors declare no conflicts of interest.

## Data Availability

The datasets generated and analyzed in this study are available from the corresponding author on reasonable request.
